# Impact of Hurricane Harvey on Healthcare Utilization and Emergency Department Operations

**DOI:** 10.5811/westjem.2020.1.41055

**Published:** 2020-04-13

**Authors:** Kimberly A. Chambers, Irfan Husain, Yashwant Chathampally, Alan Vierling, Marylou Cardenas-Turanzas, Fanni Cardenas, Kunal Sharma, Samuel Prater, Jonathan Rogg

**Affiliations:** * McGovern Medical School at UTHealth, Department of Emergency Medicine, Houston, Texas; † Emory University School of Medicine, Department of Emergency Medicine, Atlanta, Georgia; ‡ Lyndon B. Johnson Hospital, Harris Health System, Houston, Texas; § McGovern Medical School at UTHealth, Houston, Texas

## Abstract

**Introduction:**

Hurricanes have increased in severity over the past 35 years, and climate change has led to an increased frequency of catastrophic flooding. The impact of floods on emergency department (ED) operations and patient health has not been well studied. We sought to detail challenges and lessons learned from the severe weather event caused by Hurricane Harvey in Houston, Texas, in August 2017.

**Methods:**

This report combines narrative data from interviews with retrospective data on patient volumes, mode of arrival, and ED lengths of stay (LOS). We compared the five-week peri-storm period for the 2017 hurricane to similar periods in 2015 and 2016.

**Results:**

For five days, flooding limited access to the hospital, with a consequent negative impact on provider staffing availability, disposition and transfer processes, and resource consumption. Interruption of patient transfer capabilities threatened patient safety, but flexibility of operations prevented poor outcomes. The total ED patient census for the study period decreased in 2017 (7062 patients) compared to 2015 (7665 patients) and 2016 (7770) patients). Over the five-week study period, the arrival-by-ambulance rate was 12.45% in 2017 compared to 10.1% in 2016 (p < 0.0001) and 13.7% in 2015 (p < 0.0001). The median ED length of stay (LOS) in minutes for admitted patients was 976 minutes in 2015 (p < 0.0001) compared to 723 minutes in 2016 and 591 in 2017 (p < 0.0001). For discharged patients, median ED LOS was 336 minutes in 2016 compared to 356 in 2015 (p < 0.0001) and 261 in 2017 (p < 0.0001). Median boarding time for admitted ED patients was 284 minutes in 2016 compared to 470 in 2015 (p < 0.0001) and 234.5 in 2017 (p < 0.001). Water damage resulted in a loss of 133 of 179 inpatient beds (74%). Rapid and dynamic ED process changes were made to share ED beds with admitted patients and to maximize transfers post-flooding to decrease ED boarding times.

**Conclusion:**

A number of pre-storm preparations could have allowed for smoother and safer ride-out functioning for both hospital personnel and patients. These measures include surplus provisioning of staff and supplies to account for limited facility access. During a disaster, innovative flexibility of both ED and hospital operations may be critical when disposition and transfer capibilities or bedding capacity are compromised.

## INTRODUCTION

Since the 1980s, Atlantic hurricanes have increased in severity, duration, and frequency, resulting in more category IV and V hurricanes with devastating impacts.^1^ Hurricane Harvey, which made landfall on August 25, 2017, as a category IV hurricane, reached the Houston area by August 26, 2017. Over four days, one trillion gallons of rain fell over Harris County, leaving 70% of the county under at least 1.5 feet of water.^2^

Hurricane Harvey and Hurricane Katrina share the title of costliest hurricanes in United States (US) history, with the National Oceanic and Atmospheric Administration estimating $125 billion in damage.^3^ Sixty-eight deaths are directly attributable to Hurricane Harvey, of which 96% were due to flooding. A 2017 systematic review by Saulnier et al concluded that given the challenges associated with gathering data during natural disasters, information describing the relationship between health and flooding events is limited but important to understand given that about half of weather-related disasters in the last 20 years were floods.^4^

In addition to a hurricane’s direct impact on life and property, hurricanes can have important effects on healthcare operations, particularly hospital emergency departments (ED).^5^,^6^ Previous studies have reported a bimodal patient-volume surge.^7^ Pre-storm, increased patient volume may ensue in hospitals that receive patients from surrounding hospitals and facilities that face mandatory evacuation.^7^ In contrast, a relative decrease in ED patient arrival has been observed during hurricane landfall, which may be attributed to transportation difficulties during heavy rainfall and widespread flooding.^8^ Post-storm, patient volumes usually rise in affected areas and may persist, highlighting the potential for long-term impact on an ED following high-intensity hurricanes.^7^,^8^,^9^

The primary objective of this report was to describe the experience during Hurricane Harvey in narrative form at Lyndon Baines Johnson (LBJ) General Hospital’s ED, providing details of the challenges and preparation strategies that may have value for future storm events. The secondary objective was to describe patient volumes and lengths of stay (LOS) during and after Hurricane Harvey in comparison to control time periods without major storms.

## METHODS

This retrospective, mixed-methods study was approved for waiver of consent by the University of Texas Houston Health Science Center Institutional Review Board. The study and narrative reports were captured from an urban, academic county hospital with 91,000 annual visits. LBJ General Hospital is a Level 3 trauma center, which had a single intensive care unit at the time of the storm. Certain specialty services are shared with a partner county hospital. Under normal circumstances, patients in need of neurosurgery or spinal surgery, for example, are transported from LBJ to a partnering hospital.

Prior to Hurricane Harvey, LBJ was the second busiest ED in Houston. The nearest hospital with an overlapping catchment area, East Houston Regional Medical Center, closed during Hurricane Harvey due to flood damage and never reopened. LBJ absorbed many of its patients, quickly becoming the busiest ED in the city.

**Population Health Research Capsule**

What do we already know about this issue?

Studies suggest that ED patient volume falls during a major storm event and rises after. Travel to the hospital is a challenge for both patients and staff.

What was the research question?

What impact did Hurricane Harvey have on ED operations at an urban county hospital and what lessons can we learn?

What was the major finding of the study?

Advanced preparation is critical, as are flexibility and innovation in situations such as multi-day flooding and damage to hospital infrastructure.

How does this improve population health?

As flooding becomes more common in the US, anticipating the need to function independently when normal operations are impaired will protect the well being of patients and staff.

### Qualtitative Data Acquisition

We conducted semi-structured interviews with an emergency medicine (EM) faculty member and the hospital administrator who were both present in the facility during Hurricane Harvey. Three nurses, selected at random, and two residents were also interviewed and provided review to ensure consistency of recollections. To ensure accuracy we also consulted written works published immediately post-storm^10^. No variations were found.

### Quantitative Data Acquisition

LBJ’s information technology (IT) department queried the existing electronic health records (EHR) for the requested time intervals, which were a five-week time period surrounding Hurricane Harvey’s 2017 landfall, as well as control periods in 2015 and 2016. Specifically, we obtained data for the week prior to the storm, the week of the storm, and three weeks post-storm for analysis. Time periods corresponding to the same weeks of the year during 2015 and 2016 were used for comparison. We collected data on ED census (volume of visits per day), demographic details (age, race/ethnicity, gender), arrival mode (ambulance vs non-ambulance), Emergency Severity Index, ED disposition, and LOS. ED disposition was further categorized into admitted and non-admitted patients. Admitted patients included those hospitalized or transferred to another facility. Patients were classified as non-admitted if they were deceased, discharged, screened and referred, or sent to Labor and Delivery, as well as those who left against medical advice or left without being seen or completing treatment. All data points were provided in aggregate from IT from existing records; no EHR or other resources were directly searched or reviewed by research personnel.

The data set included 22,487 cases in total. Of these, 7665 cases corresponded to the 2015 study period, 7760 cases in 2016, and 7062 in 2017. Overall, the data included 105 days of observations, with 35 days corresponding to each year of study. Days of each of the included years were further ordered as values from −7 to 28, with value 0 representing the date Hurricane Harvey arrived (8/26/2017). The correspondent dates for years 2015 and 2016 were set as 8/29/15 and 8/27/16, respectively.

### Data Analysis

We reported the frequencies, measures of central tendency, and percentages for categorical variables, such as gender, ethnicity, admissions, and arrivals by ambulance per year of study. Univariate analysis was used to compare characteristics of the periods of study, with the reference period as 2016. We compared categorical variables using chi-square testing, and we analyzed continuous variables using t-tests or the Wilcoxon-Mann Whitney test if the distribution was not assumed to be normal (ie, when comparing LOS).

Additionally, we performed an analysis of covariance to understand the trends between the periods of study by using days as the unit of analysis. Data was collapsed by adding events occurring on the same day for nominal variables and by calculating the overall median of each LOS category per day. We set the period of days under study per year as independent categorical variables using days of year 2016 as reference values, while the variables of interest, such as admissions per day or arrivals by ambulance per day, were dependent variables in each analysis. A probability value of < 0.05 (two-tail) was considered statistically significant for all tests. We performed all analyses using Stata IC 15 (StataCorp LLC, College Station, TX).

## RESULTS

### Facilities and Operations

Heavy rains and high winds ravaged the areas surrounding LBJ beginning August 25, 2017, and within two days,vehicles and ambulances could no longer access the hospital, which was surrounded by six feet of water for the following five days. Patients arrived on foot and via trucks, high-water vehicles, helicopters, boats, and makeshift rafts. Once the streets surrounding the hospital became accessible, those living in communities closest to the hospital began to arrive. However, highways continued to be impassable, and hospital employees living outside the area could not relieve in-hospital personnel.

During Hurricane Harvey, LBJ sustained 250 water penetrations. Of the 179 total inpatient beds, 133 were closed secondary to damage. As a result, one of the two 16-bed bays in the ED was reallocated as an inpatient unit following approval from the State of Texas. In addition to patients seeking treatment, LBJ was inundated with displaced storm refugees seeking shelter. However, some individuals who initially only sought food and shelter also developed mediical needs since they were without necessary medications. During the five-day course, 183 storm refugees were sheltered in a large annex area separate from the ED, consuming some of the hospital’s resources.

### Notable Cases

While searching for a missing family member during the storm, a patient sustained a head injury after a fall from his all-terrain vehicle. Emergency medical services (EMS) placed him in a cervical collar but were unable to reach LBJ, instead transferring him to a dump truck for transport. Upon arrival, his head computed tomography (CT) revealed a large subdural hematoma. The ongoing storm rendered immediate EMS transport to a hospital with neurosurgical capabilities impossible, prompting hospital executive leadership to contact the Coast Guard. However, the patient’s neurological status declined rapidly, and physicians realized the Coast Guard helicopter would likely not arrive in time.

The on-call trauma surgeon had completed two years of neurosurgery training before changing surgical subspecialties. Given the concern for increasing intracranial pressure and the risk of herniation and death, it was determined that while efforts to evacuate the patient were ongoing, the surgeon would perform an emergent craniotomy. After assembling a makeshift neurosurgical tray with the necessary equipment from an array of services, the surgeon performed the first ever craniotomy at LBJ. Approximately two hours post-surgery, the Coast Guard landed at an improvised landing area, and the patient was transported to a hospital with neurosurgical capabilities where he recovered without deficits.^11^

A second notable case was that of a patient in third-degree heart block with a ventricular escape rhythm. Emergency physicians placed a transvenous pacemaker (TVP), but there were no cardiology fellows or faculty onsite to manage post-admission complications. Because LBJ does not have a coronary care unit, patients requiring a higher level of cardiac care are usually transferred—an impossible task during the immediate aftermath of the storm. Despite not routinely managing TVPs, the medical intensive care unit (MICU) cared for the patient for several days before ultimately transferring him for permanent pacemaker placement.

Most internal medicine (IM) faculty had left post-rounds on August 26, so house staff were alone on site during and post-storm, with the exception of a lone hospitalist and MICU faculty. Fortunately, power and telecommunications were never lost, so IM faculty were able to review EHRs remotely and communicate with their residents.

### ED Staffing and Basic Needs/Personal Care

At LBJ, hospital leadership executed disaster planning for hospital staff well in advance of the storm. A full complement of nurses, technicians, and ancillary staff were present for the storm ride-out and aftermath. In preparation for possible flash flooding, the hospital leadership had made arrangements early in the day on Friday, August 25, to gather and sequester ride-out teams who arrived prepared with supplies. Therefore, hospital staffing was robust during the storm’s aftermath.

In contrast, medical staffing at LBJ was less structured in its approach to disaster planning. Because it was believed there would be enough time to make arrangements once flooding materialized, early staffing contingencies were not solidified. Because the change of shifts occurred on Saturday, August 26, before the heavy rainfall ensued, faculty left the hospital, and only two EM faculty physicians were on duty when the storm began. Additional faculty who attempted to drive to the hospital encountered impassable roads.

In addition to two EM faculty, there was an EM third-year resident, two advanced practice practitioners, and two interns one month into their residency. The team’s relative inexperience meant EM faculty needed to provide a high-level of supervision while the team managed boarding and arriving patients. Sleep schedules were developed to allow 8–12 hours between periods of clinical work, but because there were only two faculty members their periods of uninterrupted rest were limited.

The hospital administrator had arranged for cots and meals for staff. However, calculations for dietary supplies were based on initial hospital census plus ride-out staffing but did not account for the storm refugees and patients who arrived. Given that supply delivery was not possible due to inaccessibility, items in the vending machines were quickly consumed. To mitigate the risk of running out of food, meal service was decreased from four to three times per day, and the hospital cafeteria switched from serving individual items to casseroles, which was a more efficient use of ingredients.

Personal care needs presented more complications for the hospital. The hospital did not have laundry service on site; hence, clean linen supplies and towels were exhausted over time. The paper scrub supply was also depleted as scrubs were given to storm refugees who were soaked from wading through flood waters. The hospital administrator authorized distribution of surplus items, such as T-shirts left over from hospital celebrations. Further, there were limited showering facilities, creating two-hour queues for those trying to shower near their units.

From outside the hospital, the ED chair worked to bring relief for staff and faculty, but many of the city’s resources were dedicated to evacuating people from flooded homes. Eventually, a high-water vehicle was designated to assist, and the water receded enough for replacement personnel to arrive after five full days.

### Patient Characteristics and Volume

Table 1 displays baseline LBJ ED volumes and patient characteristics during 2015, 2016, and 2017 over a five-week period, beginning one week prior to Hurricane Harvey. Over the full time period, the LOS for both admitted and discharged patients declined in 2017, despite a five-day boarding period during the storm. The proportion of patients arriving by ambulance was higher in 2017 when compared to 2016, although not higher than the similar time period in 2015. A better understanding of the patient volume and mode of transport differences can be gained by looking in more detail at the timeline presented in the figures.

**Figure 1:**
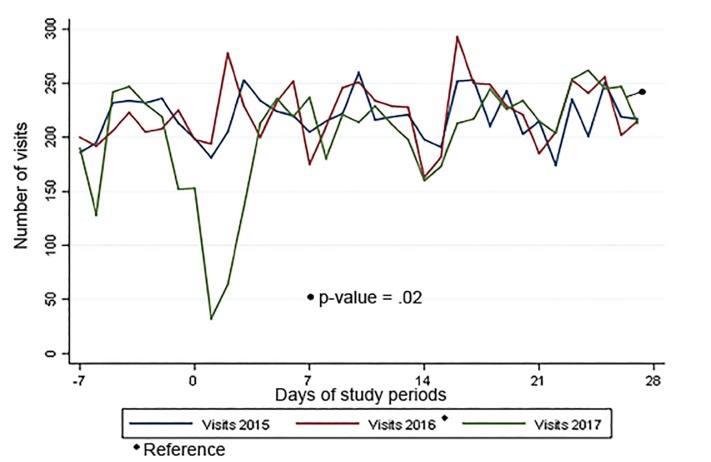
Number of visits to the Lyndon B. Johnson Hospital emergency department by period of study.

**Figure 2:**
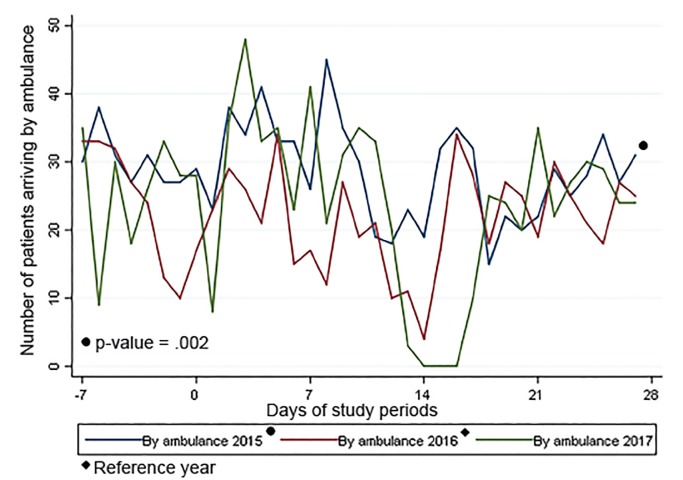
Number of patients who arrived by ambulance by period of study.

In 2017, there was a large decrease in patient volume and a corresponding increase in the percentage of patients brought in by ambulance beginning at approximately time 0, when Hurricane Harvey battered Houston (Figures 1 and 2).

When the flood waters receded post-storm, there continued to be a relative paucity of ED beds because water-damaged inpatient rooms remained closed, leading to increased ED boarding and fewer ED beds that could be used for ED throughput. Adaptive and innovative strategies were undertaken to manage having fewer beds, which will be described in greater detail in the following section.

## DISCUSSION

This is the only study we are aware of that has specifically assessed the impact of Hurricane Harvey on ED operations. Through both qualitative and quantitative assessment, we found useful data that may help other EDs prepare for a storm. Most centers experience a volume surge after a hurricane. After Hurricane Hugo (1989), ED volumes increased by 19% during the three weeks following the storm and remained high for three months,^6^ while Hurricane Andrew (1989), Hurricane Isabel (2003), and Superstorm Sandy (2012) led to even greater post-storm patient-volume surges of 35–40% in certain emergency centers.^7^,^9^,^12^ In the case of LBJ, a patient surge was not seen. Some potential patients may not have been in the area since thousands of storm refugees were housed in the city’s convention center and at other central locations farther from the hospital.

LBJ passed environmental testing to begin reopening closed inpatient beds seven weeks after the storm’s landfall. As a result, the ED adapted by transferring patients to other hospitals around or outside the city until hospital capacity normalized. Another strategy to manage fewer ED and inpatient beds was to change ED patient flow. A flex area was created, which acted as “quick care” for minor problems and housed ED patients who were awaiting the results of diagnostic testing and reassessment. Keeping patients who were not critically ill “vertical” in chairs (rather than lying in beds) was a key strategy, allowing for the treatment of a maximum number of patients who needed emergency care at LBJ.^13^

**Figure 3:**
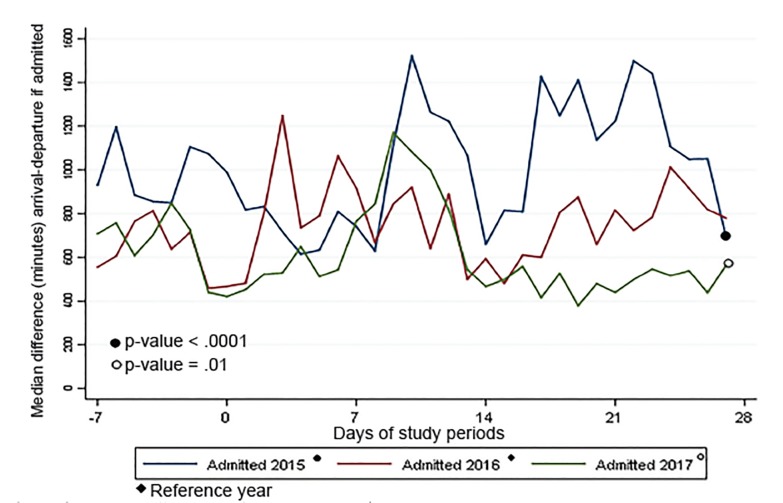
Median length of stay for admitted patients by period of study.

**Figure 4:**
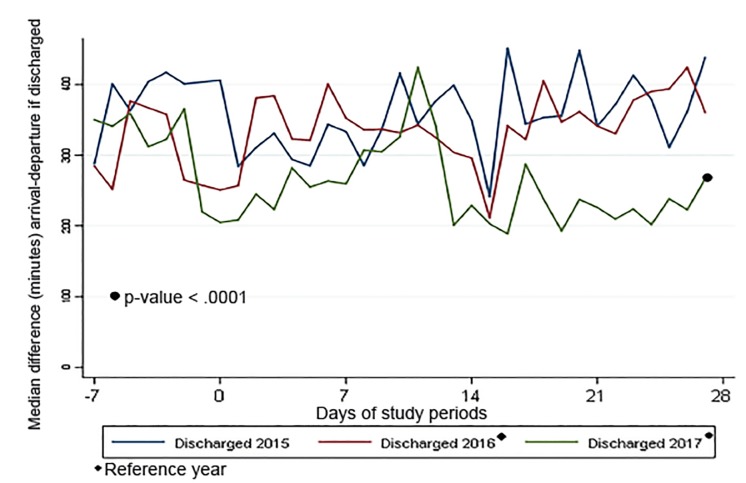
Median length of stay for discharged patients by period of study.

The processes of keeping ED patients “vertical” and initiating transfers early were associated with a lower LOS in 2017 when compared to previous years. As shown in Figure 1, approximately one week post-storm, the patient volume returned to normal, pre-storm levels. Other hospital-level changes, including postponing elective admissions and procedures, as well as a new focus on capacity management by nursing leadership, further contributed to generating capacity for ED patients. Despite having fewer available beds, Figures 3 and 4 show that approximately 10 days post-storm when changes were implemented, there was a sharp decline in patients’ median ED boarding time.

EDs and hospitals should be prepared to be self-sufficient during and directly after a severe storm, as our experience and others’ experiences have shown.^5^,^14^,^15^ Disaster planning drills have been found to be effective in preparing staff for their roles,^14^,^16^–^18^ and had there been more drills with their associated focus on pre-planning and defined policies and roles, provider staffing during the ride-out could have been considerably different.

Roads may be impassable both during and after the storm.^14^,^15^,^19^ As we saw, both EM and other providers were unable to reach the facility. Pre-disaster, consideration should be given to which subspecialists and supplies may become necessary if normal transfer processes are unavailable. In-hospital call might be needed for certain specialists, since the specialists may be unable to reach the hospital, or the hospital could endure power outages, thereby limiting communication.^14^ Early arrival times should be considered if shift/call start times coincide with the storm’s anticipated arrival so that staff members are not endangered by storm conditions. Key leadership should also consider being on site during a major storm’s ride-out for operational decision-making.^6^,^15^,^20^

Selection of personnel for anticipated prolonged duty in an environment requiring adaptability and personal discomfort should be part of hospital disaster planning. Accounting for staff well being is crucial when planning for multi-day disasters, as performing duties outside of normal routine is common.^21^ Overall, work shifts should be centered on safety and staff endurance.^6^,^15^,^21^ Therefore, staffing decisions should consider providers’ need for rest, meaning that more providers than usual will likely be required to be in-house to sustain a safe work rotation.

Arrangements for sleeping quarters and provisioning of food, water, and other unique needs should be established in advance. When considering supplies, administrators should base estimates accounting for storm refugees who may arrive at the hospital for shelter. In the wake of a disaster, estimating food and water provisions is challenging given that supplies will probably be needed for more individuals than those originally accounted for.^14^,^15^,^21^,^22^

Individuals should consider ways that they can be self-sufficient in the event that conditions require it. Bringing non-perishable food, bottled water, personal hygiene items, personal medications, and sleeping gear may be helpful.^14^,^15^,^21^ The Harris Health System, which includes LBJ Hospital, has a disaster plan that outlines that its employees are responsible for bringing personal items, including food and water, when participating in a hurricane ride-out. However, as evidenced by the depletion of vending machines and the rationing of food for patients, refugees, and staff, additional supplies brought by employees were not enough for the duration of the storm. Following Hurricane Katrina, a post-storm survey revealed that staff at Charity Hospital did not fully understand that bringing a seven-day supply of food was their responsibility.^21^ As only 20% of responders indicated that they understood this responsibility, Brevard et al recommend that details of personal responsibilities, such as bringing personal supplies, should be reinforced annually when refreshing employees on disaster-planning protocols and performing disaster drills.^21^

Disaster planners should also recognize that staff may themselves be victims of the storm.^15^,^19^,^23^ During Hurricane Harvey, one member of the ED staff lost a son. Another member of the housestaff had to arrange the rescue of his wife and family while trapped at LBJ. These occurrences led the hospital administrator to recommend that a mental health counselor be part of the ride-out team to support staff and patients who are in need, which has been suggested in previous literature.^19^,^24^,^25^

## LIMITATIONS

This report has several important limitations. The qualitative data that is presented in narrative form is retrospective in nature; therefore, interviews are subject to recall bias. Wherever possible, the providers’ accounts were verified by multiple sources, including articles written closer to the time of the event. A larger sampling of staff could also have been useful to provide additional data for qualitiative analysis.

The analyzed data were also collected retrospectively. While investigators attempted to match the storm’s time period to similar ones in prior years by selecting the same days of the week and season of the year, it is possible that other time periods would have been more representative. Further, it may have been of use to track a longer timeline than five weeks, since disaster conditions in the city continued for much longer. Prospective data collection would be preferred, with details on the challenges recorded during and immediately after the storm.

## CONCLUSION

Early and thorough preparation by leadership and individual team members can alleviate some stresses that result from being on duty in the ED during severe storm and flooding events. Both the hospital and the medical staff should have defined disaster-operations procedures in place before a storm event, with goals of being self-sufficient on both a facility and individual level and of having excess resources on site. Our experience of water damage and decreased accessibility to the hospital was similar to what other facilities have described. We did not experience patient-volume surges that other studies have reported; however, we had a relative surge in the ED volume since many beds were occupied by non-ED patients and normal admission processes were curtailed. Despite bed closures, ED LOS and boarding decreased relative to similar time periods, which may have been due to the dynamic operational changes and the shifting of resources initiated by the administration team during the disaster. Flexibility and innovation are key in adapting ED operations to overcome the challenges created by disaster conditions.

## Supplementary Information



## Figures and Tables

**Table 1:** Comparison of differences in patient’s characteristics, length of stay, and boarding time for years 2015 and 2017, with 2016 as the reference year.

Variable	2015 8/22–9/25	P-value	2016 8/20–9/23 (Reference)	P-value	2017 8/19–9/22
Total patients	7665		7760		7062
Age	41.1 (18.5)	0.002	42.0 (17.8)	<0.0001	43.13 (18.32)
Ethnicity		<0.0001		0.85	
Hispanic	4236 (55.9)		4595 (59.8)		4137 (59.41)
Non-Hispanic	3346 (48.9)		3088 (40.2)		2827 (40.59)
Arrival by ambulance, n (%)		<0.0001		<0.0001	
Yes	1009 (13.7)		772 (10.1)		844 (12.45)
No	6331 (86.3)		6900 (89.9)		5936 (87.55)
ESI, mean (SD)	2.9 (0.72)	< 0.0001	3.0 (0.75)	0.40	3 (0.77)
ED disposition, n (%)		0.09		0.43	
Admitted/observation	1216 (15.9)		1154 (14.9)		1023 (14.49)
Discharged	6448 (84.1)		6606 (85.1)		6038 (85.51)
ED LOS: admitted patients, median minutes (IQR)	n = 1216 976 (606–1490)	< 0.0001	n = 1154 723 (481–1099)	< 0.0001	n = 1023 591 (412–953)
ED LOS: discharged patients, median minutes (IQR)	n = 6448 356 (226–543)	< 0.0001	n = 6604 336 (216–505)	< 0.0001	n = 6036 261 (157–412)
Boarding time: admitted or observation, median minutes (IQR)	n = 1183 470 (211–921)	< 0.0001	n = 1120 284 (142–554)	< 0.001	n = 974 234.5 (117–508)
Boarding time: discharged patients, median minutes (IQR)	n = 5731 22 (11–42)	0.22	n = 5782 23 (12–40)	0.10	n = 5544 22 (12–39)

*ESI*, Emergency Severity Index; *ED*, emergency department; *LOS*, length of stay; *IQR*, interquartile range; *SD*, standard deviation.
