# Supplementary material for: Impact of Hurricane Harvey on Healthcare Utilization and Emergency Department Operations
Source: West J Emerg Med. 2020 Apr 13;21(3):586–94. doi: 10.5811/westjem.2020.1.41055 (PMC7234707; doi:10.5811/westjem.2020.1.41055)
Supplement: Supplementary file 1 [file wjem-21-586-s001.docx]

**Supplemental Images**


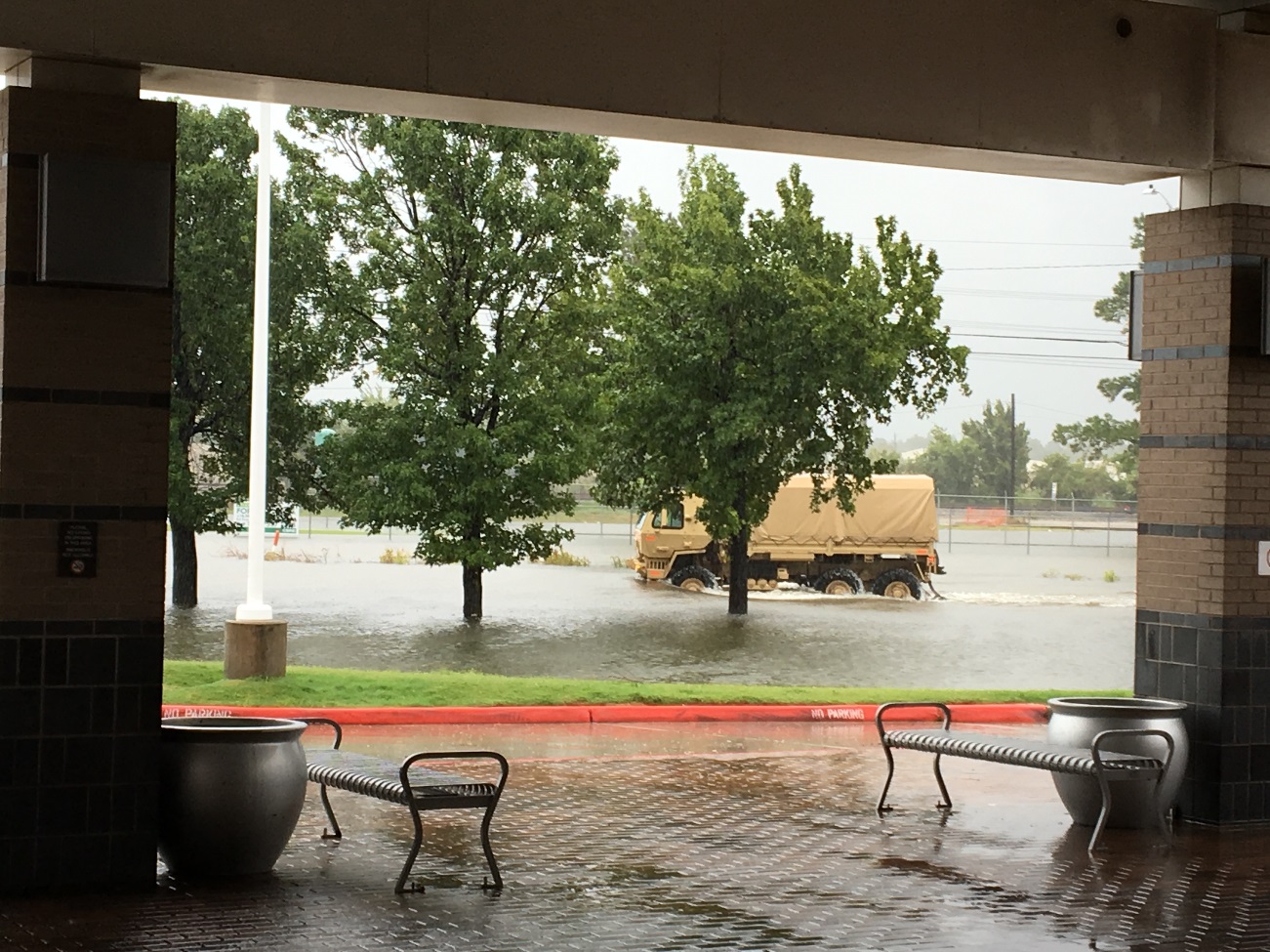


**Image 1.** An image of one of the military vehicles that patrolled the neighborhood and brought storm evacuees to LBJ. Photo courtesy of Harris Health System.


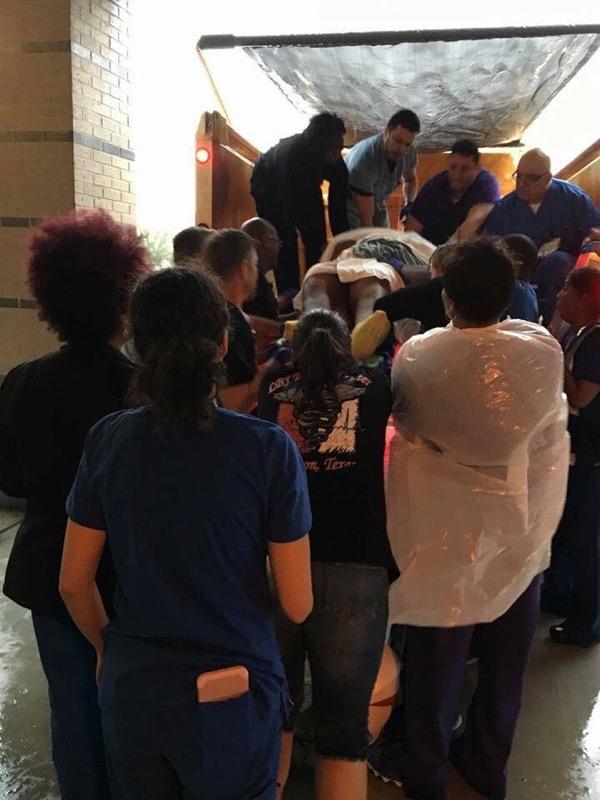


**Image 2.** Patient arriving by truck with LBJ Emergency Center (EC) team moving the patient into the EC.
Photo courtesy of C. Rene Fenner.
